# Prediction of Walnut Moisture Content Using Impact Acoustics, Physical Dimensions, and Machine Learning

**DOI:** 10.3390/foods15172951

**Published:** 2026-08-22

**Authors:** Aref Sepehr, Maciej Zaborowicz, Francesco Marinello, Lorenzo Guerrini

**Affiliations:** 1 Department of Land, Environment, Agriculture and Forestry (TESAF), Università Degli Studi di Padova, Viale Dell’Università 16, 35020 Legnaro, PD, Italy; aref.sepehr@phd.unipd.it (A.S.); francesco.marinello@unipd.it (F.M.); 2 Department of Biosystems Engineering, Poznan University of Life Sciences, Wojska Polskiego 50, 60-627 Poznań, Poland; maciej.zaborowicz@up.poznan.pl; 3 Institute of Computing Science, Poznan University of Technology, Piotrowo 2, 60-965 Poznań, Poland

**Keywords:** walnuts, moisture content, ML, acoustic, WSTD, GBM, RF

## Abstract

Walnuts are commercially important tree nuts whose moisture content (MC) influences quality, shelf life, and post-harvest processing. This study evaluated the potential of low-cost acoustic sensing combined with machine learning for non-destructive MC prediction. Sixty in-shell walnuts were subjected to controlled drying at 40 °C for 26 h, with acoustic recordings and physical measurements collected every two hours. Acoustic signals were processed using Wavelet Soft Threshold Denoising (WSTD), Short-Time Fourier Transform (STFT), and Variational Mode Decomposition (VMD), and features were extracted from the resulting signals. Predictive models included generalized linear models (GLM), random forests (RF), gradient boosting machines (GBM), and Partial Least Squares (PLS) approaches. Following grouped walnut-level validation, the highest MC prediction performance was achieved by the model combining dimensional and acoustic descriptors (RF: *R*^2^ = 0.836; GBM: *R*^2^ = 0.826), while the model combining drying time and acoustic descriptors achieved moderate predictive performance (RF: *R*^2^ = 0.762; GBM: *R*^2^ = 0.760). Overall, the results provide proof-of-concept evidence that acoustic descriptors may complement physical measurements for non-destructive walnut moisture-content prediction. However, substantially larger independent datasets collected across multiple cultivars, production batches, acquisition conditions, and external validation studies will be required before practical industrial implementation can be considered.

## 1. Introduction

Walnuts (*Juglans regia* L.), widely cultivated across America, Asia, and Europe, have both ecological benefits and economic potential. Walnuts are recognized as one of the four major tree nuts, alongside almonds, cashews, and hazelnuts. The walnut kernel contains essential nutrients and bioactive compounds with strong antioxidant properties. However, due to limitations in post-harvest processing, particularly in moisture control, appropriate storage conditions are critical for preserving kernel quality [1,2,3]. Drying itself is a crucial step in walnut processing, since water content strongly influences walnut quality; accurate moisture measurement is vital for effective drying [4].

Traditional detection methods, such as manual inspection, vibration sifting, and wind separation, have significant limitations. Manual inspection is destructive, time-consuming, and labor-intensive, while mechanical methods are energy-intensive, less environmentally friendly, and may damage the shell, making them unsuitable for internal quality assessment [5]. The food industry increasingly requires precise and efficient inspection methods. Conventional techniques are often insufficient, creating demand for intelligent technologies, including machine learning (ML) applied to impact sound analysis and image-based evaluation of physical parameters [6]. Some studies have examined nut dimensions and related properties, e.g., moisture content (MC) [7,8], whereas others have investigated acoustic analysis as a promising non-destructive tool for quality evaluation and crack detection [3,6,9,10].

Recent studies have expanded the application of non-destructive sensing technologies for nut-quality assessment. Acoustic emission analysis has been successfully used to classify pistachios according to shell status using convolutional neural networks [11], while hyperspectral imaging combined with machine-learning algorithms has demonstrated strong potential for moisture-content prediction in macadamia nuts [12]. Dimensional and morphological descriptors have also proven valuable for evaluating nut quality traits [8]. In parallel, studies investigating moisture-related phenomena, including moisture migration during walnut drying [13] and sound absorption behaviour in wood-based materials [14], have provided useful insights into the relationship between moisture and material properties. Despite these advances, integrated approaches combining physical and acoustic information for moisture-content assessment remain limited.

Acoustic-based methods have gained increasing attention for non-destructive food quality assessment. Impact acoustic sensing, in which samples are dropped onto a surface to produce measurable sound responses, has been widely applied. Extracted signals are typically analyzed using methods, e.g., Fast Fourier Transform (FFT), time-domain analysis, Mel spectrograms, or EEMD. A range of ML models, including Support Vector Machines (SVM), Least Squares SVM (LSSVM), Extreme Learning Machines (ELM), Random Forest (RF), Gradient Boosting Machine (GBM), Multilayer Perceptron (MLP), CNN, and Partial Least Squares Regression (PLSR), have been used in agriculture and food sector to classify or predict parameters such as MC and shell integrity. Optimization algorithms, including the Arithmetic Optimization Algorithm (AOA), Particle Swarm Optimization (PSO), and Gray Wolf Optimization (GWO), are often employed to enhance performance, while different validation methods, such as Leave-One-Out (LOO) cross-validation, help ensure model generalizability [3,5,9,10,11,15,16,17].

A persistent challenge in post-harvest walnut processing is the non-destructive evaluation of MC during drying. To the best of our knowledge, few previous studies have integrated acoustic features with physical dimensional measurements for moisture-content prediction during dynamic walnut drying. Furthermore, many existing studies employ controlled laboratory acquisition systems that may be difficult to translate directly into industrial processing environments. To address these gaps, this study proposes a simple, minimal-equipment method for assessing walnut quality. By analyzing sound features and physical dimensions of walnuts at different drying stages, the study evaluates their combined potential for dynamic moisture-content monitoring. The objective is to evaluate the feasibility of integrating drying time, physical dimensions, and impact-acoustic descriptors for non-destructive MC prediction during controlled walnut drying.

## 2. Materials and Methods

### 2.1. Sample Preparation

This study builds upon our previous preliminary work on sound evaluation of walnuts [17]. In-shell walnut samples (*Juglans regia* L.) were collected from Azienda Agricola Bellombra (Adria, Rovigo, Italy) after dehulling and before drying, then transported to the laboratory and stored in plastic bags at 4 °C until experimentation; ambient laboratory temperature was 26 ± 4 °C. For this study, 60 fresh walnuts of the Lara cultivar were randomly selected and labeled. The experimental design generated repeated measurements from the same walnuts across multiple drying stages and impact surfaces. Consequently, although 1334 valid acoustic recordings were obtained, the effective biological sample size remained 60 walnuts. The repeated measurements were intentionally collected to characterize moisture evolution within individual walnuts during the drying process rather than to increase the number of independent biological samples. The weight and three principal shell dimensions (length, width, and thickness) of each in-shell walnut were measured before drying. Following the dimensional definitions used for walnut morphology by Ercisli et al. (2012) [18], length was defined as the maximum pole-to-pole dimension, width (cheek diameter) as the maximum transverse dimension, and thickness (suture diameter) as the maximum dimension perpendicular to the width. Dimensional measurements were obtained using the same manual caliper and standardized orientation for all samples, while weight was measured using BIL counting scales (LAUMAS Elettronica, Montechiarugolo, Italy) with a maximum load of 6 kg and a precision of 0.1 g. Each shell dimension was measured once per walnut; repeated measurements were not performed, and therefore no replicate average was calculated. Because these measurements were obtained only before drying, length, width, and thickness were treated as baseline descriptors of external shell geometry rather than as measurements of dynamic dimensional shrinkage. Walnut weight, in contrast, was recorded at each sampling interval to monitor moisture loss throughout the drying process. A dynamic drying process was carried out in a Binder FED 53-UL forced-convection oven (BINDER GmbH, Tuttlingen, Germany) at 40 °C for 26 h, without increasing the drying temperature, to preserve walnut quality. Periodic weighing at regular intervals of 2 h was performed to monitor moisture reduction, and the impact sound of the samples was recorded, as explained in the following section. Each measurement required the temporary removal of samples from the oven for approximately 3 to 4 min, after which they were immediately returned. Periodic interruption during gravimetric drying experiments is commonly adopted in food drying kinetics studies and is considered acceptable when exposure duration is limited [19,20]. Moisture reduction during drying, described by Fick’s law (Equation (1)), affects both parameters.

(1) $$\frac{\partial X}{\partial t}=D_{e}\times \left[\frac{\left(\partial^{2}X\right)}{\left({\partial r}^{2}\right)}+(\frac{2}{r}).(\frac{\partial X}{\partial r})\right]$$

where X is the local MC, r is the radial distance, t is time, and $D_{e}$ is the effective moisture diffusivity [21,22].

At the end of the drying process, walnuts were further dried at 103 °C for approximately 24 h to determine the final dry mass of each walnut, which served as the reference value for moisture-content calculations in wet-basis MC (Equation (2)).

(2) $$MC(\%)=\left[\frac{\left(W1-W2\right)}{W1}\right]\times 100$$

where W1 and W2 represent the initial and dry masses of the samples, respectively [23].

For each walnut, the moisture content at every sampling interval was calculated using the walnut mass measured at that specific drying time point and the final dry mass obtained after oven drying at 103 °C until constant weight. Consequently, each acoustic recording was associated with a time-specific MC value derived from the corresponding walnut mass trajectory throughout the drying process.

### 2.2. Recording Conditions

**Figure 1:**
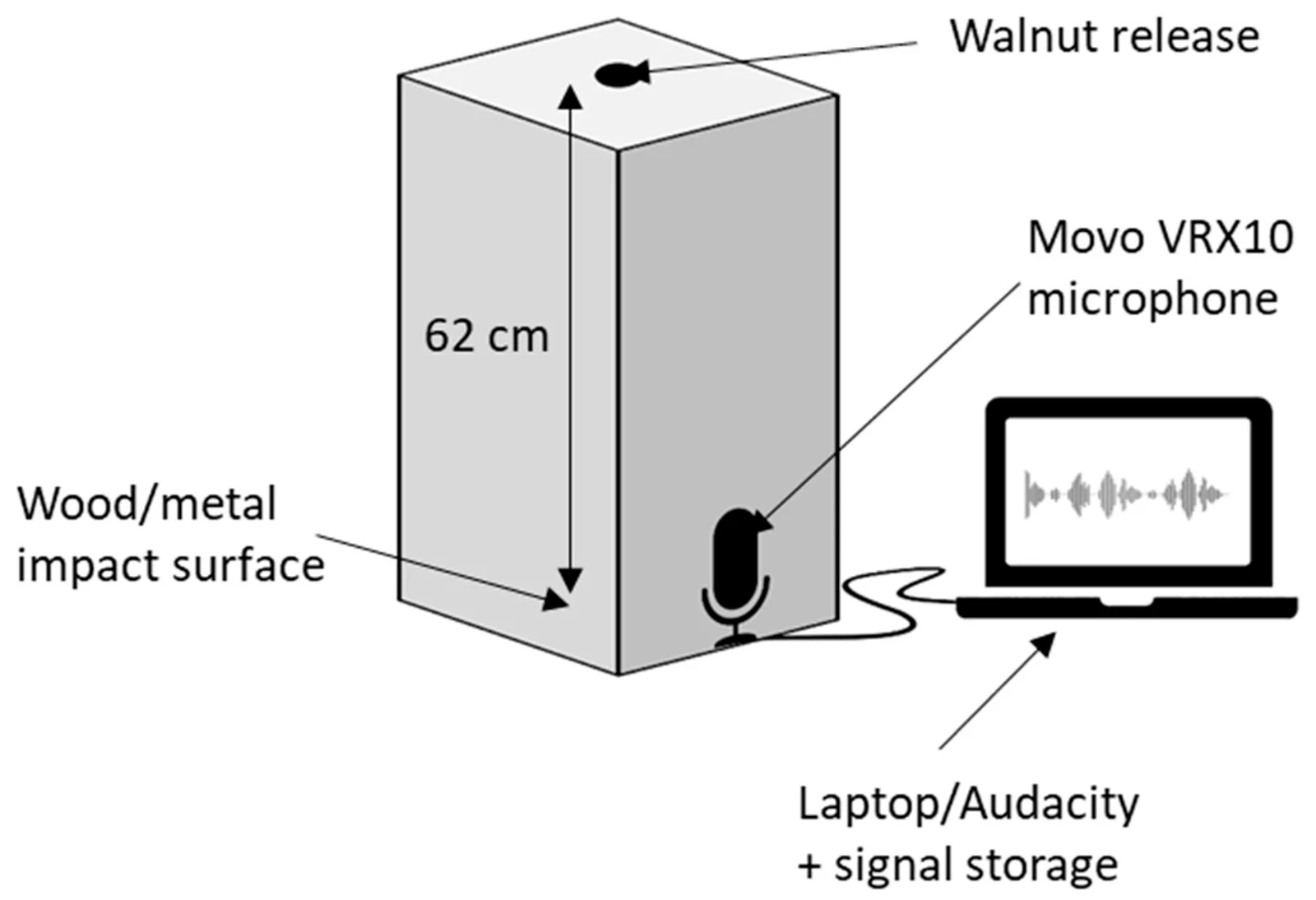
Experimental setup used for walnut impact-sound acquisition showing the release height (62 cm), interchangeable impact surface (wood or metal), microphone position near the impact point, acquisition computer, and overall signal acquisition workflow.

To collect acoustic data, two custom soundproof boxes (internal dimensions of 60 × 37 × 32 cm) were designed and constructed in the laboratory. To isolate the impact sound as the main variable, the interior surfaces of the boxes were lined with acoustic foam, except for the impact surface. One box incorporated a wooden impact surface and the other a metal surface, chosen specifically to produce distinct sound characteristics upon walnut impact and provide a broader range of acoustic data for model training. In both setups, a hole was designed in the top lid to allow walnuts to be manually dropped onto the impact surface from a fixed height of 62 cm. At the same time, a microphone inside the box captured the resulting impact sounds directly near the impact point (Figure 1). A Movo VXR10 microphone (Movo Photo Group LLC, Shenzhen, China), with a frequency response of 35 Hz to 18 kHz, was used for sound acquisition. Recordings were collected with the free version of Audacity^®^ software, Audacity 3.7.0 (Muse Group, Limassol, Cyprus), connected to a laptop for real-time monitoring and storage.

Impact sound measurements were performed on 60 walnuts across 13 time points, recorded at 2 h intervals over the 26 h drying period, and on two distinct impact surfaces per sample. A theoretical maximum of 1560 acoustic observations was expected (60 samples × 13 time points × 2 surfaces). However, due to complete breakage of some samples during drying and impact testing, the final dataset comprised 1334 valid recordings. These recordings should not be interpreted as statistically independent biological observations because multiple recordings originated from the same walnut throughout the drying process. Recordings from both surfaces were pooled to increase the acoustic variability associated with the different impact-surface properties. The impact-surface type was therefore not included as an independent predictor in the present models, because the objective was to evaluate model performance in the presence of surface-related acoustic variability rather than to compare wood and metal as separate experimental factors. The descriptive statistics of the fresh walnut physical dimensions and weight before the drying procedure are presented in Table 1.

### 2.3. Sound Feature Extraction

All data handling, feature extraction, and model training were performed in Python (version 3.14.5), a standard platform for scientific computing and ML, owing to its flexibility and broad library support. The Librosa library was employed for advanced audio processing and feature extraction, while scikit-learn was used for statistical analysis and the implementation of ML algorithms. These tools provided a reliable and reproducible framework for managing the resulting dataset [24].

To achieve the research objectives and ensure robust feature extraction and model development, the workflow included systematic preprocessing of raw acoustic signals, extraction of relevant sound and physical features, and application of ML models for prediction. Preprocessing began with wavelet soft threshold denoising (WSTD), impact detection, and variational mode decomposition (VMD) applied to the raw audio signals. Subsequently, sound features such as Mel spectrograms, spectral descriptors, Mel-frequency cepstral coefficients (MFCCs), delta MFCCs, and higher-order statistics (e.g., skewness and kurtosis of root mean square (RMS), Auto-correlation Energy (AE), Zero-crossing rate (ZCR)) were extracted. These sound features, combined with physical parameters (thickness, length, and width), were analyzed using regression approaches with generalized linear model (GLM), RF, and GBM. The preprocessing operations were applied sequentially as a single signal-processing pipeline rather than evaluated as independent processing alternatives.

#### 2.3.1. Noise Reduction

In this work, Mel spectrograms, spectral, MFCCs, delta MFCCs, Skewness, and Kurtosis for RMS, AE, ZCR, etc., have been used for evaluation. We first applied noise reduction to raw audio files using WSTD because of its proven effectiveness in handling nonstationary, short-duration signals while preserving important information. Compared to hard thresholding, WSTD provides better continuity and prevents signal distortion caused by coefficient discontinuities during reconstruction, improving the overall efficiency of signal denoising [25]. The function of the soft threshold is shown in Equation (3).

(3) $${\hat{Y}}_{\left(j,k\right)}=\left\{\begin{array}{ll} \mathrm{sgn}\left(Y_{\left(j,k\right)}\right)\left(\left|Y_{\left(j,k\right)}\right|-\lambda \right), & \left|Y_{\left(j,k\right)}\right|\geq \lambda \\ 0, & \left|Y_{\left(j,k\right)}\right|<\lambda \end{array}\right.$$

where ${\hat{Y}}_{\left(j,k\right)}$ is the denoised coefficient, $Y_{\left(j,k\right)}$ is the processed wavelet coefficients at scale j, index k, λ refers to the threshold, sgn is the sign function [26,27].

The threshold parameter *λ* was selected according to standard wavelet denoising practices reported in previous studies involving transient acoustic and nonstationary signals. Preliminary exploratory evaluations were performed to ensure adequate noise suppression while preserving the transient characteristics of walnut impact signals. Soft thresholding was preferred over hard thresholding because it provides smoother signal reconstruction and reduces discontinuities in the reconstructed waveform. The objective of the denoising stage was not algorithmic optimization itself, but rather the stabilization of acoustic feature extraction under heterogeneous recording conditions [25,28].

#### 2.3.2. Impact Detection

Since sounds were obtained under real-life conditions, background noise reduction was necessary to ensure consistency. To address this, and following the WSTD, an impact detection technique was applied. To isolate the acoustic response generated by falling walnuts, we implemented an energy-spectral fusion approach for sound event localization. Audio files were segmented into overlapping frames of 1024 samples (≈23.2 ms at 44.1 kHz) with a hop length of 512 samples, and short-time energy was computed within each frame. In parallel, a Short-Time Fourier Transform (STFT) (Equation (4)) with the same parameters was performed to calculate spectral flux, capturing frame-to-frame changes in spectral magnitude.

(4) $${STFT}_{x(t)}\left(m,w\right)=\int_{-\infty }^{+\infty }x\left(t\right)w\left(t-mT\right)e^{-jwt}dt$$

where x(t) is the input signal, *m* and w are the frame index and angular frequency, respectively, w(t−mT) is the window function centered at time mT, $e^{-jwt}$ is the complex exponential for frequency analysis, and dt is the differential element, representing integration over time [29,30].

The two metrics were then combined into a unified detection score, and the frame with the highest score, representing the most probable impact moment, was identified. Around this peak, a fixed-length segment of 1.0 s (±0.5 s) was extracted, unless the signal was shorter, in which case the window length was adaptively adjusted. This procedure is consistent with the findings of previous studies [31,32] showing that combining time and frequency-domain cues improves the detection of short-duration sound events in complex acoustic environments. Similarly, Ma et al. (2024) [33] highlighted the effectiveness of energy-based methods for transient acoustic localization, emphasizing their robustness and simplicity in low Signal-to-Noise Ratio (SNR) conditions. By integrating amplitude and spectral flux, the proposed method provides efficient, unsupervised segmentation of impact sounds with minimal assumptions and without the need for deep learning models or multi-sensor systems.

#### 2.3.3. Signal Decomposition

To further analyze the short transient acoustic signals, Variational Mode Decomposition (VMD) was employed because of its robustness in decomposing nonstationary and noise-prone signals into band-limited intrinsic mode functions (IMFs). In the present study, the decomposition parameters were selected based on configurations commonly reported in previous VMD studies involving transient acoustic and vibration signals [26,34,35]. Preliminary exploratory evaluations were additionally conducted to verify decomposition stability and preservation of the dominant impact-related waveform characteristics.

The number of modes was fixed at K=5 to achieve a balance between sufficient signal decomposition and avoidance of over-fragmentation of the acoustic response, shown in Equation (5). Lower *K* values resulted in insufficient separation of transient components, whereas higher values introduced redundant low-energy oscillatory modes. The penalty parameter was set to α = 2000 and the convergence tolerance to 10^−7^, following stable configurations reported in related literature [34,35]. The objective of this stage was not the optimization of VMD itself, but rather the extraction of representative acoustic components suitable for downstream feature analysis and machine-learning evaluation under simplified industrial simulation conditions.

(5) $${min}_{\left\{uk\right\},\left\{wk\right\}}=\left\{\sum_{k=1}^{k}{\left\|\partial_{t}\left[\left(\delta \left(t\right)+j\pi t\right)*u_{k}\left(t\right).e^{-iw_{k}t}\right]\right\|}_{2}^{2}\right\},\;s.t\sum_{k=1}^{k}u_{k}=f(t)$$

where f(t) is the original signal, $u_{k}(t)$ is the kth mode to be extracted, and $u_{k}$ and $w_{k}$ are modes with center frequency, ∗ denotes convolution, δ(t) is the unit impulse function, j is the imaginary unit, and $\partial_{t}$ is the time derivative [26,35].

Regularized VMD (RVMD), despite its ability to suppress oscillatory behavior, due to its higher computational complexity, has not been chosen for this work. As explained in Liu et al. (2024) [26], RVMD introduces L1 regularization to control IMF smoothness. However, this advantage is more critical in noisy, nonlinear, or chaotic signals, which were not predominant in our recordings, as noise suppression was already addressed through WSTD and impact detection. Following VMD decomposition, a Mutual Information (MI)-based selection method was applied to identify the most relevant IMF for feature extraction. Each IMF, together with the original signal, was uniformly resampled to 100 points to ensure dimensional consistency. MI regression was then used to compute the MI score between each IMF and the original signal, with the MI value expressed in Equation (6). The IMF with the highest score, representing the greatest shared information, was selected for subsequent analysis.

(6) $$MI\left(X,Y\right)=H\left(Y\right)-H\left(Y|X\right)$$

In this case, MI measures the amount of shared information between an IMF X and the original signal Y by calculating the difference between the entropy of Y and the conditional entropy of Y given X [36].

#### 2.3.4. Sound Features

In this study, a comprehensive set of acoustic features was extracted to characterize the impact signals generated by dropping walnuts during dynamic drying, as summarized in Table 2. ZCR and RMS were included to capture temporal frequency and amplitude [3]. Skewness and kurtosis were calculated to describe waveform asymmetry and peakedness, following their successful application in postharvest nut classification [11]. Power spectral density (PSD) was computed to evaluate the distribution of acoustic energy across frequencies, providing insights into internal structure. In addition, perceptual spectral features were extracted, including Mel spectrograms, MFCCs, delta MFCCs, and spectral contrast, which have been reported in several studies as effective descriptors for capturing differences in acoustic feature patterns [10,11,37].

These dimensionalities were chosen based on established conventions in acoustic signal processing and optimized for capturing spectral and temporal variations while maintaining computational tractability. All features were extracted using the Librosa-based feature extraction pipeline. The distribution of acoustic predictors across feature categories is reported in Supplementary Table S1.

To describe the distribution and variability of extracted acoustic parameters, descriptive statistics of representative time- and frequency-domain features were computed across all valid recordings (*n* = 1334). Due to the high dimensionality of extracted acoustic features, only representative statistics from some features are presented in Table 3.

### 2.4. Modeling Framework

Four predictive configurations were progressively developed for MC estimation, as summarized in Table 4. The initial analysis focused on exploring the relationship between drying time and MC using a GLM. The dataset was split into 80% for training and 20% for testing subsets, and this split was maintained consistently across all models to ensure comparability of performance metrics. Drying time was considered essential for evaluation and monitored in all steps, as it represents the primary variable continuously monitored in industrial practice and directly relates to moisture reduction according to Fick’s second law (Equation (1)). This framework reflects dynamic monitoring conditions in which sequential measurements are available during processing. The GLM served as a baseline to assess predictive accuracy under idealized conditions.

This baseline framework was progressively expanded in Models 2, 3, and 4 to include real-world variations in nut size, acoustic features, and their combination. The GLM was selected for its simplicity and clarity, making it a suitable reference for comparison. It assumes normally distributed residuals and applies the link function described in Equation (7).

(7) $$g\left[E\left(Y\right)\right]=\beta_{0}+\beta_{1}X_{1}+\beta_{2}X_{2}+\cdots +\beta_{p}X_{p}$$

where g is a link function, E(Y) is the expected value of the response variable Y, and $\beta_{0},\;\beta_{1},\;\ldots ,\;\beta_{p}$ are the model parameters (coefficients) associated with the predictor variables $X_{1},\;X_{2},\;\ldots ,\;X_{p}$ [38].

The final acoustic feature matrix included temporal (ZCR, RMS, and AE statistics), spectral descriptors, Mel spectrogram coefficients, MFCCs, delta MFCCs, and spectral contrast features, resulting in a total of 150 extracted acoustic predictors used for the subsequent machine learning analyses. The distribution of predictors across feature categories is reported in Supplementary Table S1. No explicit feature-selection procedure was applied prior to model training. The objective of the present study was to evaluate the predictive capability of the complete acoustic representation rather than identify a minimal subset of descriptors. RF and GBM can accommodate correlated predictors and high-dimensional feature spaces; however, this capability does not eliminate the risk of unstable model performance or overfitting when the number of independent biological samples is limited. Accordingly, the present RF and GBM analyses are interpreted as exploratory proof-of-concept models rather than as evidence of a fully optimized or broadly generalizable predictive framework. To provide a dimensionality-reduction benchmark, PLSR was additionally implemented in the revised analysis. For exploratory interpretation, RF- and GBM-based feature importance was also examined for M.3, and the 10 highest-ranked acoustic predictors are reported in Supplementary Table S3.

In these extensions, Model 2 for MC prediction (M.2) incorporated size-related parameters (length, width, and thickness). Model 3 for MC (M.3) added acoustic features. Finally, Model 4 for MC (M.4) combined both size and acoustic features, creating the most comprehensive predictor set and allowing the joint contribution of dimensional and acoustic information to be evaluated. To support the predictive framework, two ensemble-based ML algorithms, RF and GBM, were implemented in regression modes. RandomForestRegressor and GradientBoostingRegressor were applied for MC prediction. These algorithms were chosen for their ability to handle high-dimensional data and capture both linear and nonlinear interactions with minimal preprocessing. Model evaluation was revised using grouped train/test splitting based on walnut identity to avoid data leakage arising from repeated measurements of the same walnut. Consequently, all recordings associated with a given walnut, including different drying times and impact surfaces, were assigned exclusively to either the training or test subset. The 80/20 grouped partition therefore corresponded to 48 independent walnuts in the training subset and 12 independent walnuts in the test subset. Repeated recordings were treated as measurements nested within these biological units and were not counted as independent samples.

For moisture prediction models, all available drying stages were considered. The grouped splitting strategy ensured that recordings from the same walnut never appeared simultaneously in the training and testing subsets, thereby providing a more rigorous estimation of model generalization performance. In addition to RF and GBM, Partial Least Squares Regression (PLSR) was implemented as a baseline chemometric approach for moisture prediction. The optimal number of latent components was selected within the training subset using grouped cross-validation.

The RF model consisted of an ensemble of 100 decision trees (n_estimators = 100), trained using the bagging strategy [39]. Each tree was trained on a bootstrap sample of the data and used a random subset of features, improving robustness and reducing variance. Final predictions were obtained by averaging the outputs of all trees (Equation (8)).

(8) $$\hat{y}RF\left(x\right)=(\frac{1}{B})\times \sum_{b=1}^{B}fb(x)$$

where $\hat{y}RF$ is the predicted value for input x using the RF, B is the total number of trees, and fb(x) is the prediction of $b^{th}$ decision tree. The randomization and averaging mechanisms of RF can reduce variance compared with individual decision trees [40,41]; however, they do not eliminate the risk of overfitting, particularly when the predictor space is large relative to the number of independent samples. RF models are nonparametric and capture feature interactions through the hierarchical structure of decision trees [39].

The GBM builds trees sequentially, where each new tree attempts to correct the residual errors of the combined previous trees [40]. The GBM was configured with 100 estimators (n_estimators = 100) and the default learning rate of 0.1. The prediction function at iteration m is defined as Equation (9).

(9) $$F_{m}\left(x\right)=F_{\left(m-1\right)}\left(x\right)+y_{m}h_{m}(x)$$

where $F_{m}(x)$ is the ensemble prediction at iteration m, $F_{\left(m-1\right)}(x)$ is the ensemble prediction from the previous iteration, $h_{m}(x)$ is the base learner (a decision tree) trained to predict residuals, and $y_{m}$ learning rate at iteration m. This stage-wise optimization allows GBM to focus learning on difficult-to-predict samples and refine predictions over iterations [42,43]. Similar to RF, the GBM relies on decision trees and does not include hidden layers in the neural network sense, with complexity controlled by the number of trees, their depth, and the learning rate [39].

### 2.5. Statistical Metrics

In this study, the predictive performance of regression models (GLM, RF, and GBM) was evaluated using a set of widely recognized statistical indicators: Mean Squared Error (MSE), Root Mean Squared Error (RMSE), Mean Absolute Error (MAE), Coefficient of Determination (*R*^2^), Mean Squared Percentage Error (MSPE), Root MSPE (RMSPE), and Mean Absolute Percentage Error (MAPE) (Equations (10)–(16)). These indicators capture both absolute and relative prediction errors, providing complementary perspectives on model robustness, bias, and variance across datasets [44,45].

(10) $$MSE=\frac{1}{n}\sum_{i=1}^{n}{{(y}_{i}-{\hat{y}}_{i})}^{2}$$

(11) $$RMSE=\sqrt{\frac{1}{n}\sum_{i=1}^{n}{{(y}_{i}-{\hat{y}}_{i})}^{2}}$$

(12) $$MAE=\frac{1}{n}\sum_{i=1}^{n}\left|y_{i}-{\hat{y}}_{i}\right|$$

(13) $$R^{2}=1-\left[\frac{\sum_{i=1}^{n}{{(y}_{i}-{\hat{y}}_{i})}^{2}}{\sum_{i=1}^{n}{{(y}_{i}-{\bar{y}}_{i})}^{2}}\right]$$

(14) $$MSPE=\frac{100}{n}\;\sum_{i=1}^{n}{\left(\frac{y_{i}-{\hat{y}}_{i}}{y_{i}}\right)}^{2}$$

(15) $$RMSPE=\sqrt{\frac{100}{n}\;\sum_{i=1}^{n}{\left(\frac{y_{i}-{\hat{y}}_{i}}{y_{i}}\right)}^{2}}$$

(16) $$MAPE=\frac{100}{n}\;\sum_{i=1}^{n}\left|\frac{y_{i}-{\hat{y}}_{i}}{y_{i}}\right|$$

where $y_{i}$ is the actual observed value and ${\hat{y}}_{i}$ is the predicted value for the *i*^th^ sample, $\hat{y}$ is the mean of actual observed values, and n represents the number of observations (sample size).

To further assess the statistical reliability of these performance metrics, bootstrap-based confidence intervals (CI) were estimated for MAE, RMSE, and *R*^2^ using 1000 resampling iterations with replacement applied to the grouped test-set predictions. The 95% confidence intervals were obtained from the 2.5th and 97.5th percentiles of the empirical bootstrap distributions. The bootstrap estimation does not require assumptions about the underlying distribution, making it particularly advantageous in cases of limited sample size or skewed error distributions. The resulting confidence intervals offer insight into the variability of model performance and enable more reliable comparisons between models and configurations.

These metrics were applied to evaluate the ML performance of models such as GLM, RF, and GBM. Many studies have employed the same statistical indicators for assessing model accuracy across diverse domains, including the food industry, engineering, signal processing, etc. [26,35,44,45,46], which affirms the reliability and comparability of these indicators in regression-based prediction scenarios.

## 3. Results and Discussion

**Figure 2:**
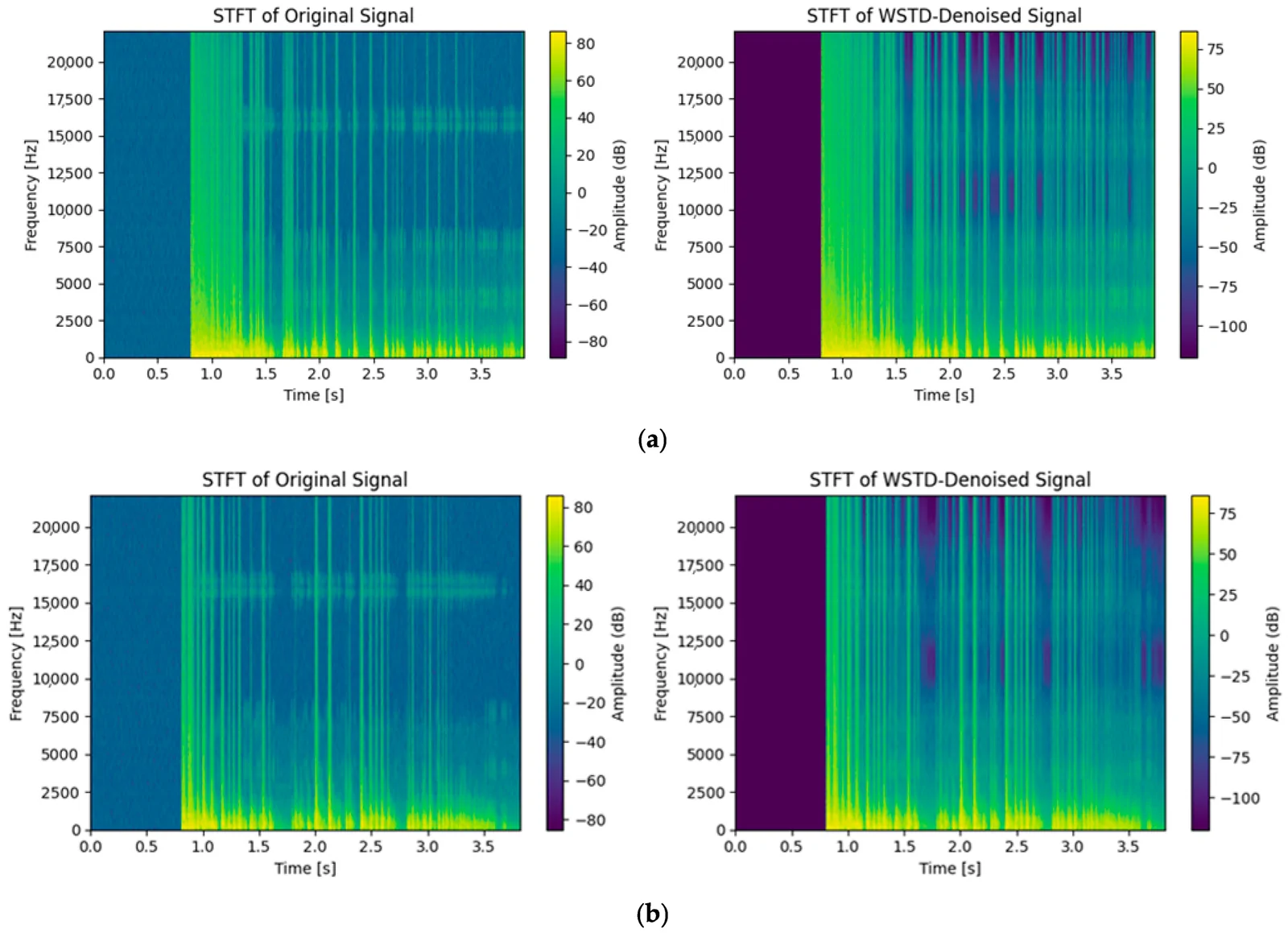
Comparison of the denoised signal and time-frequency spectrum of the wavelet soft threshold denoising (WSTD)-denoised signal, showing clearer impact-related energy and reduced background noise after Short-Time Fourier Transform (STFT). (**a**) is the fresh and (**b**) is the same dried sample.

**Figure 3:**
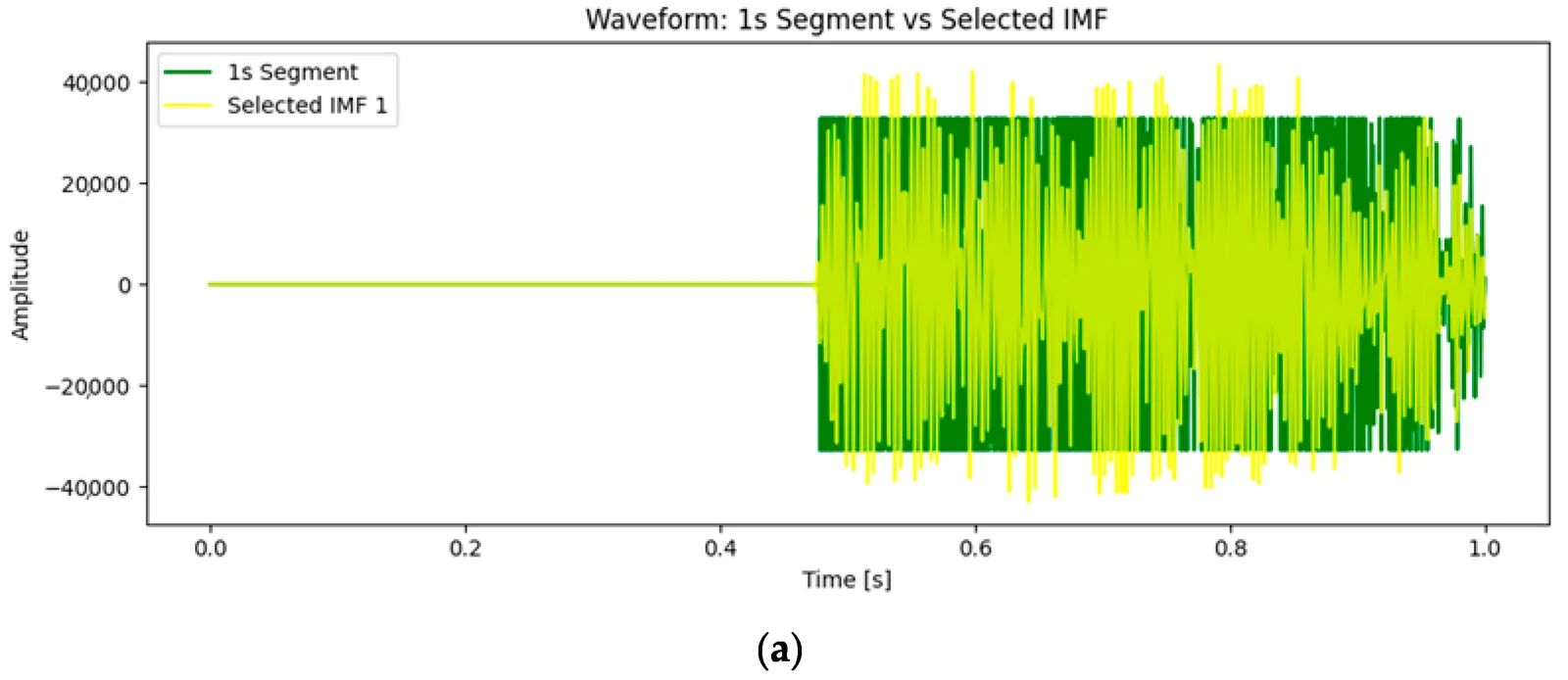

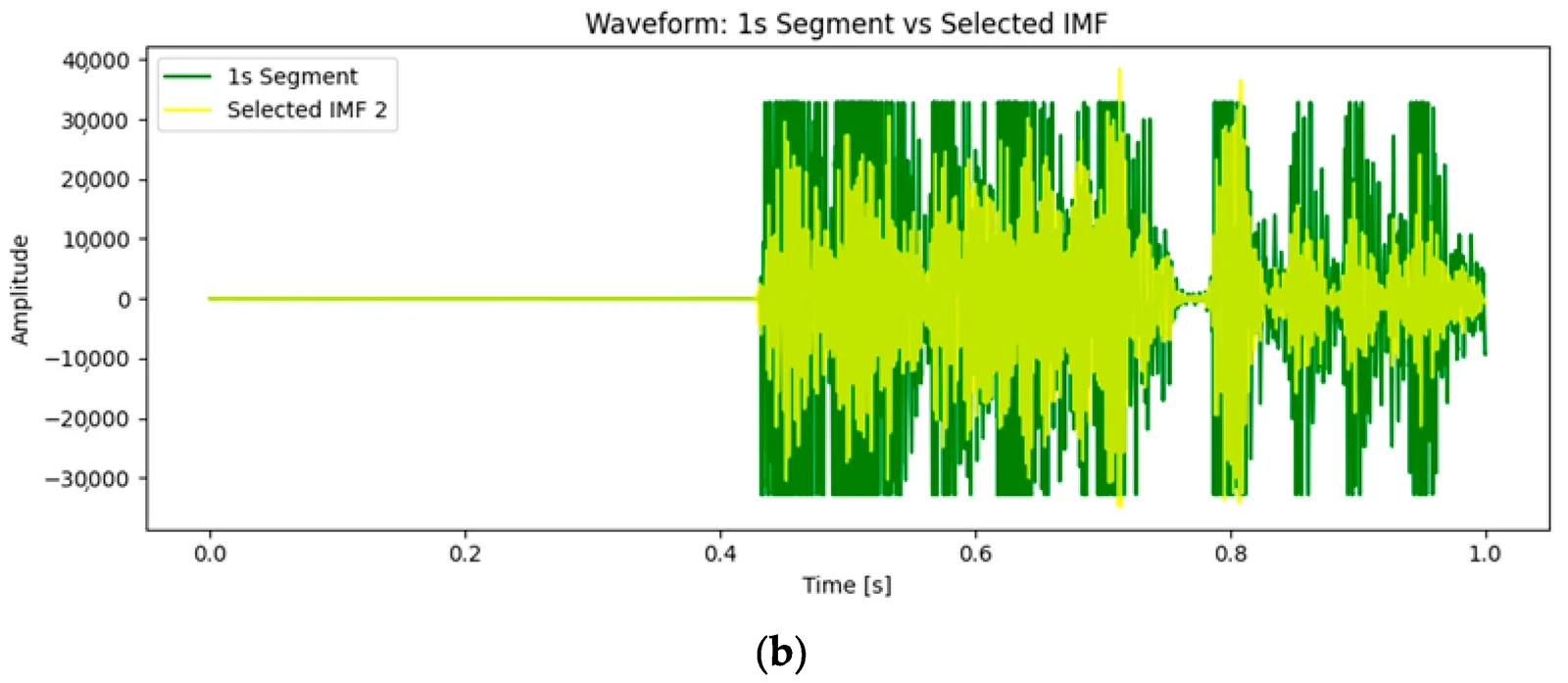
Segment versus selected intrinsic mode function (IMF) (time domain), waveform of the 1 s impact segment compared to the selected IMF, showing strong alignment in signal shape and key features. (**a**) is the fresh and (**b**) is the same dried sample.

**Figure 4:**
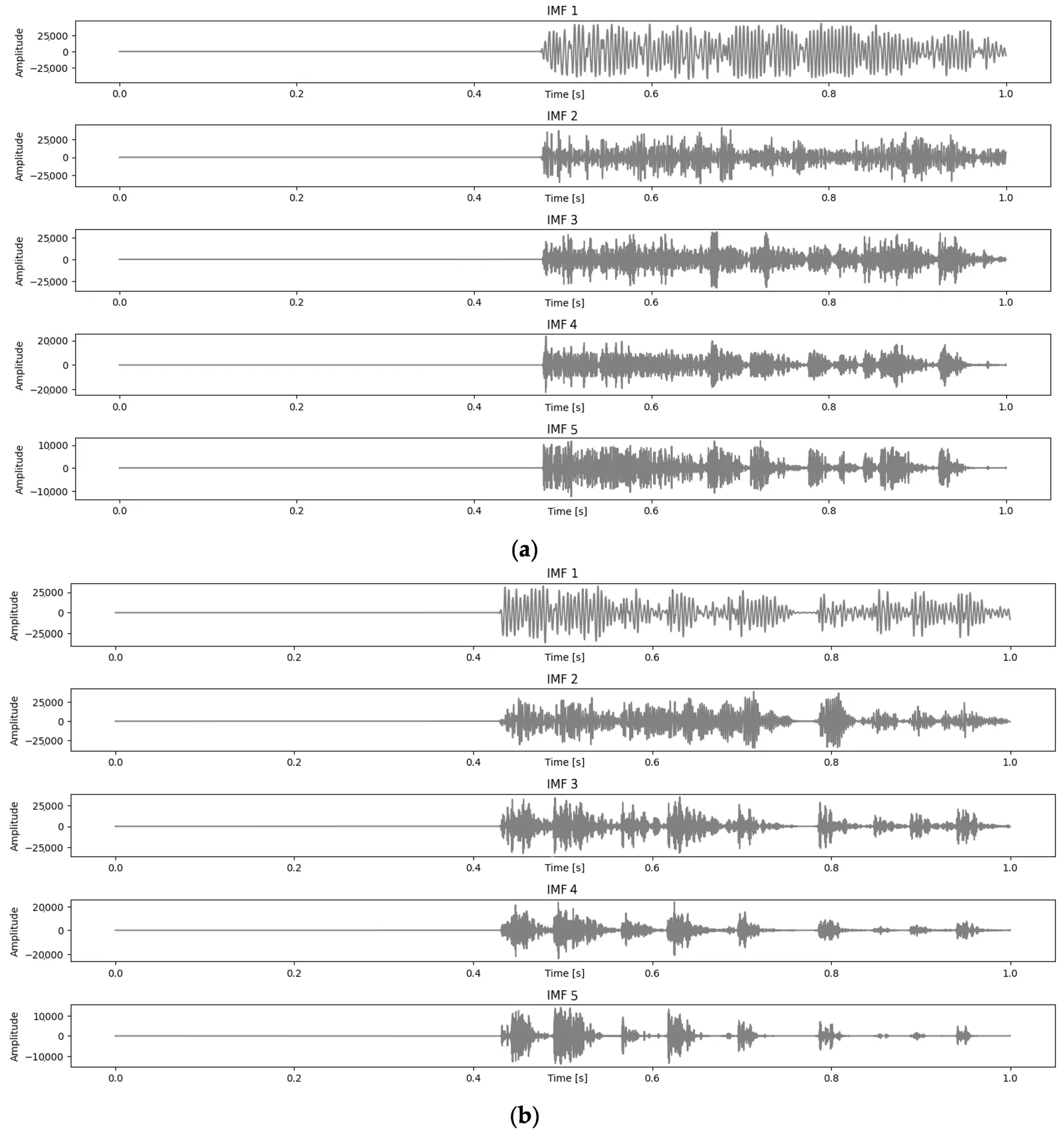
IMFs 1–5 of the decomposed segment capture a distinct oscillatory mode from the impact segment, highlighting dominant frequency content. (**a**) is the fresh sample and (**b**) is the same dried one.

Pre-processing results are illustrated in Figure 2, Figure 3 and Figure 4. The STFT of the WSTD-denoised signal showed a more concentrated distribution of spectral energy around the impact event, particularly in the mid-frequency range. Compared with the original signal, the denoised signal exhibited a lower noise floor and reduced energy dispersion, indicating that WSTD preserved the main transient characteristics while suppressing non-stationary noise. Similar effects of wavelet-based denoising on transient signals have been reported in previous studies [37,47]. Figure 2 illustrates this behavior for a representative walnut in the fresh state and after 26 h of drying.

Building upon this, the comparison between the 1 s impact segment and the selected IMF reveals high temporal consistency. The selected IMF captures the dominant shape and envelope of the impact event while filtering out finer noise fluctuations. This confirms the effectiveness of MI-based selection in isolating the most informative IMF from the VMD output, enabling feature extraction to focus on structurally relevant components of the signal, as demonstrated by Shen et al. (2021) [48] in their denoising framework. This is illustrated in Figure 3, showing a sample both fresh and after 26 h of drying.

A closer examination of the first five IMFs extracted via VMD reveals a clear progression in frequency content and structural contribution to the original impact segment. IMF 1 captures the highest-frequency oscillations and primarily reflects sharp transients or residual noise components. IMF 2 continues to represent high-frequency content but with slightly more structured waveform features. IMF 3 emerges as the most informative component, closely aligning with the amplitude envelope and temporal shape of the impact signal, indicating that it retains the dominant physical characteristics of the event. IMF 4 transitions into lower-frequency behavior, capturing broader waveform features likely associated with the decaying phase or resonant response following the initial impact. Lastly, IMF 5 exhibits smooth, low-frequency oscillations with minimal amplitude variation, suggesting it contributes to the signal’s overall baseline or underlying trend. These modes provide a hierarchical representation of the acoustic event, from fine transient details to global structural patterns, confirming observations from previous VMD-based studies [26,35]. Representative results for fresh and dried samples are reported in Figure 4.

Finally, the ML evaluation of predictive models for MC is summarized in Table 5 after grouped walnut-level validation. RF generally achieved the highest predictive performance across the evaluated models, followed closely by GBM [49]. The strongest predictive accuracy was obtained for M.4, which combined dimensional and acoustic descriptors, indicating that the integration of multiple information sources improved moisture estimation performance [15]. Bootstrap-derived 95% confidence intervals for MAE, RMSE, and *R*^2^ are reported in Supplementary Table S2.

After implementing grouped walnut-level validation, RF consistently achieved the highest predictive performance across the moisture prediction models; this performance of RF has also been shown in different applications [44]. The baseline temporal model (M.1), based exclusively on drying time, achieved an *R*^2^ of 0.707. Incorporating walnut dimensional parameters substantially improved prediction accuracy, with Model M.2 reaching an *R*^2^ of 0.814 using RF.

The inclusion of the time-plus-acoustic model (M.3) demonstrated that sound-derived descriptors contain relevant information associated with walnut moisture dynamics, although the predictive performance remained lower than that obtained using dimensional parameters alone. The best overall performance was obtained for M.4, which combined temporal, dimensional, and acoustic descriptors, achieving *R*^2^ = 0.836, RMSE = 3.22, and MAE = 2.30 using RF. Previous studies have also shown that increasing model complexity can improve predictive performance [15,50]. Exploratory feature-importance analysis of M.3 showed that drying time remained the dominant predictor in both RF and GBM. Among the acoustic descriptors, the highest-ranked features varied between algorithms; however, contrast_1_mean, contrast_2_mean, mel_3_mean, mfcc_9_std, and mfcc_11_std appeared among the 10 highest-ranked acoustic predictors in both models. These rankings are reported in Supplementary Table S3 and should be interpreted descriptively rather than as stable feature-selection results because of the number of independent walnuts and the high-dimensional predictor space.

PLSR baseline models generally achieved lower predictive performance than RF and GBM, particularly for more complex feature combinations, indicating the presence of nonlinear relationships among acoustic descriptors, dimensional characteristics, and moisture evolution. These results suggest that walnut dimensions constitute the most informative predictor group for moisture estimation, while acoustic descriptors provide complementary information that further improves prediction when combined with dimensional measurements. For RF, adding the acoustic descriptors to the model containing drying time and dimensional measurements increased *R*^2^ from 0.814 to 0.836, corresponding to an absolute improvement of 0.022; therefore, the incremental contribution of the acoustic feature set should be regarded as modest under the present experimental conditions. Although the acoustic feature space was relatively high-dimensional, the inclusion of PLSR provided an explicit latent-variable benchmark for comparison. The superior performance of RF and GBM suggests that nonlinear relationships among acoustic descriptors and physical measurements contribute meaningfully to moisture prediction and that predictive performance was not solely driven by dimensionality reduction. These findings are in line with previous research on different ML applications [16,51].

Although the dimensional parameters showed strong predictive performance, future studies should further investigate the integration of acoustic sensing with computer vision approaches under real industrial conditions.

Environmental noise and the use of different impact surfaces, such as wood and metal, in this study, may have influenced the results by introducing variability that models struggled to capture. In addition, the computational cost of feature extraction must be considered. Therefore, future studies should emphasize robustness across varieties, moisture levels, factory noise, varied surface conditions, and in situ impact or excitation methods, potentially incorporating signal denoising, data augmentation, domain adaptation, or physics-aware modeling to improve generalization.

Although literature-supported parameter configurations and preliminary exploratory evaluations were adopted for WSTD and VMD, the present study did not aim to perform exhaustive optimization of signal decomposition parameters. Future studies should systematically evaluate the influence of decomposition settings, threshold selection, and alternative denoising approaches on feature robustness and model generalizability.

Several limitations should be acknowledged. Despite the fact that grouped walnut-level validation substantially reduced the risk of data leakage among repeated measurements, the study remains limited by the relatively small number of biological samples and the use of a single experimental acquisition setup. Although bootstrap confidence intervals provide an estimate of the variability associated with the reported performance metrics, they do not compensate for the limited number of independent biological samples and should not be interpreted as evidence of model generalizability. Furthermore, the dimensional measurements (length, width, and thickness) were obtained only before drying and therefore served as baseline descriptors of external shell geometry rather than measurements of dynamic dimensional shrinkage. Because each shell dimension was measured only once, operator-related measurement repeatability and the associated measurement uncertainty could not be quantified. Future studies including repeated dimensional measurements during drying could determine whether shrinkage dynamics provide additional predictive information. In addition, the acoustic predictor space remained substantially larger than the number of independent biological samples, and future studies should investigate feature-selection strategies, sparse modelling approaches, substantially larger independent walnut populations collected from multiple production batches and cultivars, repeated grouped validation strategies, and external validation datasets to further improve model robustness and generalizability. Additionally, because WSTD, STFT-assisted impact localization, VMD, and MI-based IMF selection were applied successively within a single processing workflow, the present analysis does not isolate the independent contribution of each preprocessing stage. Future studies should include systematic ablation analyses to quantify the individual contribution of signal-processing operations and acoustic feature families. Moreover, the study was conducted using a single walnut cultivar subjected to controlled drying conditions and standardized impact testing. Although this experimental design ensured consistency during data collection, the resulting models should not be assumed to generalize directly to other cultivars, drying protocols, storage conditions, or industrial processing environments without additional validation.

## 4. Conclusions

This study demonstrated the feasibility of integrating machine learning and signal-processing techniques for the non-destructive prediction of walnut moisture content (MC). By combining traditional physical parameters (e.g., dimensions and drying time) with acoustic features extracted from impact sounds, the proposed framework achieved promising predictive performance. The use of WSTD and STFT enhanced signal quality while preserving transient impact characteristics, whereas VMD combined with MI-based IMF selection enabled the extraction of acoustically relevant signal components. Among the regression models, the highest predictive performance was achieved by models incorporating dimensional measurements, while acoustic descriptors provided complementary information that further improved prediction when combined with physical parameters.

Overall, the findings provide proof-of-concept evidence that acoustic descriptors contain complementary information for non-destructive walnut moisture-content assessment when integrated with physical measurements. Nevertheless, the present results should not be interpreted as evidence of a fully generalizable predictive framework. Additional validation using substantially larger independent walnut populations, multiple cultivars, different production batches, diverse acquisition conditions, and external validation datasets will be necessary before practical industrial deployment can be established. Future research should also investigate multi-sensor approaches, including computer vision and sensor fusion strategies, to improve robustness and generalizability.

## Figures and Tables

**Table 1:** Descriptive statistics of initial physical dimensions and weight of walnut samples (*n* = 60).

Variable	Mean	SD	Min	Max
Weight (g)	17.69	4.05	7.96	28.35
Length (mm)	35.45	1.94	30.51	40.41
Width (mm)	37.48	2.18	30.97	42.30
Thickness (mm)	35.34	1.94	29.02	38.98

**Table 2:** List of sound features extracted from the audio files.

Feature Name	Type	Description
zcr_mean, zcr_std, zcr_skew, zcr_kurt	Temporal features	Zero Crossing Rate (ZCR) statistics. ZCR measures how frequently the signal changes sign, reflecting noisiness or percussiveness. Mean = average ZCR, Std = variability, Skew = asymmetry, Kurt = peakedness of distribution.
rms_mean, rms_std, rms_skew, rms_kurt	Temporal/Energy features	Root Mean Square (RMS) energy statistics. RMS measures the short-term signal power. Mean = overall energy, Std = variability, Skew = asymmetry of energy distribution, Kurt = sharpness or flatness of energy distribution.
ae_mean, ae_std, ae_skew, ae_kurt	Spectral features	Auto-correlation Energy (AE) statistics. Represents the temporal periodicity and harmonicity of the signal. Mean = average periodic energy, Std = variability, Skew = distribution shape, Kurt = peakedness.
sc_mean	Spectral features	Spectral Centroid mean indicates the “center of mass” of the spectrum, related to perceived brightness.
sro_mean	Spectral features	Spectral Roll-off mean, frequency below which a fixed percentage (e.g., 85–95%) of the spectral energy is contained. Relates to spectral shape and sharpness.
sf_mean	Spectral features	Spectral Flux mean measures the frame-to-frame change in the spectrum, reflecting temporal variation and onsets.
psd_mean, psd_std	Spectral features	Power Spectral Density (PSD) statistics. PSD describes how signal power is distributed over frequency. Mean = average spectral energy, Std = variability across frequencies.
mel_1_mean, …, mel_40_mean mel_1_std, …, mel_40_std	Mel spectrogram	Statistics of Mel-scaled spectrogram coefficients (40 filterbanks). Represent spectral energy in perceptually motivated frequency bands. Mean = average energy per band, Std = variability.
mfcc_1_mean, …, mfcc_13_mean mfcc_1_std, …, mfcc_13_std	MFCC	Mel-Frequency Cepstral Coefficient statistics (MFCCs) (typically first 13). Capture spectral envelope (timbre) characteristics. Mean = average cepstral value, Std = variability.
delta_mfcc_1_mean, …, delta_mfcc_13_mean	Delta MFCCs	First-order derivatives of MFCCs. Capture temporal dynamics of the spectral envelope. Mean values describe overall trends in cepstral changes.
contrast_1_mean, …, contrast_7_mean contrast_1_std, …, contrast_7_std	Spectral contrast	Measures the difference between spectral peaks and valleys in sub-bands (usually 7). Describes the distribution of energy between harmonic and non-harmonic components. Mean = average contrast per band, Std = variability.

**Table 3:** Descriptive statistics of representative time-domain and frequency-domain acoustic features extracted from impact recordings (*n* = 1334).

Variable	Mean	SD	Min	Max
zcr_mean	0.01	0.03	0.00	0.51
rms_mean	5622.44	2674.46	208.77	18,799.95
ae_mean	4286.86	2251.25	166.14	16,114.26
psd_mean	3847.84	2904.71	4.31	20,062.56

**Table 4:** Columns M.1, M.2, M.3, and M.4 specify which variables have been used in Model 1, Model 2, Model 3, or Model 4, respectively, and MC% represents the dependent variable used as the target for moisture evaluation.

Variable (MC% Prediction)	M.1	M.2	M.3	M.4
hours (drying times)	X			
hours + SZ (size = thickness, width, and length)		X		
hours + SD (sound features)			X	
hours + SD + SZ				X

**Table 5:** Validation performance metrics.

Model	Algorithm	RMSE	MAE	*R* ^2^
M.1	GLM	4.31	3.30	0.707
M.2	RF	3.43	2.41	0.814
M.2	GBM	3.50	2.61	0.807
M.2	PLSR	3.59	2.68	0.797
M.3	RF	3.88	2.72	0.762
M.3	GBM	3.91	2.73	0.760
M.3	PLSR	4.20	3.12	0.722
M.4	RF	3.22	2.30	0.836
M.4	GBM	3.32	2.45	0.826
M.4	PLSR	3.68	2.74	0.787

## Data Availability

The original contributions presented in this study are included in the article/Supplementary Materials. Further inquiries can be directed to the corresponding author.

## Supplementary Materials

The following supporting information can be downloaded at: https://www.mdpi.com/article/10.3390/foods15172951/s1, Table S1: Distribution of the extracted acoustic predictors across feature categories; Table S2. Bootstrap-derived 95% confidence intervals for regression model performance metrics after grouped walnut-level validation; Table S3. Top 10 acoustic predictors ranked by RF and GBM feature importance for the time-plus-acoustic model (M.3).
