# Supplementary material for: Prediction of Walnut Moisture Content Using Impact Acoustics, Physical Dimensions, and Machine Learning
Source: Foods. 2026 Aug 22;15(17):2951. doi: 10.3390/foods15172951 (PMC13564734; doi:10.3390/foods15172951)
Supplement: Supplementary file 1 [file foods-15-02951-s001.zip › foods-4461052-supplementary.pdf]

Supplementary Materials

Table S1. Distribution of the extracted acoustic predictors across feature categories.

Table S2. Bootstrap-derived 95% confidence intervals for regression model performance metrics after grouped walnut-level validation.

Table S3. Top 10 acoustic predictors ranked by RF and GBM feature importance for the time-plus-acoustic model (M.3).

To further explore the contribution of walnut size to MC prediction, an

Table S1. Distribution of the extracted acoustic predictors across feature categories.

Feature category	Number of predictors	Description
Zero-Crossing Rate (ZCR) statistics	4	Mean, standard deviation, skewness, and kurtosis of the zero-crossing rate
Root Mean Square (RMS) statistics	4	Mean, standard deviation, skewness, and kurtosis of signal energy
Auto-correlation Energy (AE) statistics	4	Mean, standard deviation, skewness, and kurtosis of autocorrelation energy
Spectral descriptors	5	Spectral centroid, spectral roll-off, spectral flux, and power spectral density statistics
Mel spectrogram features	80	Mean and standard deviation of 40 Mel-frequency filter-bank coefficients
Mel-Frequency Cepstral Coefficients (MFCCs)	26	Mean and standard deviation of 13 MFCC coefficients
Delta MFCCs	13	Mean values of the first-order derivatives of 13 MFCC coefficients
Spectral contrast features	14	Mean and standard deviation of seven spectral contrast bands
Total acoustic predictors	150	Complete acoustic feature space used for model development

Table S2. Bootstrap-derived 95% confidence intervals for regression model performance metrics after grouped walnut-level validation.

Model	Algorithm	RMSE (95% CI)	MAE (95% CI)	R^2 (95% CI)
M.1	GLM	4.31 (3.86–4.78)	3.30 (2.98–3.65)	0.707 (0.66–0.75)
M.2	RF	3.43 (3.03–3.84)	2.41 (2.13–2.79)	0.814 (0.77–0.85)
M.2	GBM	3.50 (3.12–3.85)	2.61 (2.34–2.88)	0.807 (0.76–0.84)
M.2	PLSR	3.59 (3.17–4.02)	2.68 (2.40–2.97)	0.797 (0.76–0.83)
M.3	RF	3.88 (3.48–4.28)	2.72 (2.41–3.04)	0.762 (0.71–0.80)

M.3	GBM	3.91 (3.45–4.35)	2.73 (2.41–3.07)	0.760 (0.71–0.80)
M.3	PLSR	4.20 (3.72–4.69)	3.12 (2.80–3.47)	0.722 (0.67–0.77)
M.4	RF	3.22 (2.87–3.56)	2.30 (2.03–2.56)	0.836 (0.80–0.87)
M.4	GBM	3.32 (2.93–3.73)	2.45 (2.18–2.73)	0.826 (0.79–0.86)
M.4	PLSR	3.68 (3.21–4.14)	2.74 (2.45–3.03)	0.787 (0.75–0.82)

Table S3. Top 10 acoustic predictors ranked by RF and GBM feature importance for the time-plus-acoustic model (M.3).

Algorithm	GBM		RF	
Rank	Acoustic feature	Importance	Acoustic feature	Importance
1	mel_3_mean	0.004841	ae_std	0.003121
2	contrast_2_mean	0.003698	contrast_2_mean	0.003116
3	mfcc_13_mean	0.003267	mfcc_9_std	0.002913
4	mel_1_mean	0.002894	contrast_3_mean	0.002879
5	mel_18_std	0.002832	mel_3_std	0.002463
6	mfcc_11_std	0.002706	delta_mfcc_3_mean	0.002432
7	mfcc_9_std	0.002414	mel_3_mean	0.002432
8	contrast_7_mean	0.002281	delta_mfcc_1_mean	0.002405
9	contrast_1_mean	0.002259	mfcc_11_std	0.002327
10	mfcc_7_mean	0.002073	contrast_1_mean	0.002296
